# Efficacy of the Combination of Voriconazole and Caspofungin in Experimental Pulmonary Aspergillosis by Different *Aspergillus* Species

**DOI:** 10.1007/s11046-013-9719-z

**Published:** 2013-12-06

**Authors:** Ming Zhang, Xin Su, Wen-Kui Sun, Fei Chen, Xiao-Yong Xu, Yi Shi

**Affiliations:** Department of Respiratory and Critical Care Medicine, Jinling Hospital, Nanjing University School of Medicine, 305 East Zhongshan Road, Nanjing, 210002 China

**Keywords:** Voriconazole, Caspofungin, Combination, *Aspergillus fumigatus*, *Aspergillus flavus*, *Aspergillus niger*

## Abstract

**Objectives:**

Invasive pulmonary aspergillosis (IPA) caused by *Aspergillus fumigatus*, *Aspergillus flavus*, or *Aspergillus niger* is associated with high mortality. We evaluated the efficacy and compared the therapeutic effect differences of voriconazole (VRC) in combination with caspofungin (CAS) in transiently neutropenic rats infected by *A. fumigatus, A. flavus*, or *A. niger.*

**Methods:**

Treatment groups consisted of VRC (10 mg/kg q12 h) monotherapy, CAS (1 mg/kg/day) monotherapy, combination of VRC (10 mg/kg q12 h) + CAS (1 mg/kg/day), and no drug for 10 consecutive days. The efficacy and the difference in the treatments were evaluated through prolongation of survival, reduction in serum galactomannan levels and residual fungal burden, and histological studies.

**Results:**

For all the strains, the combination of VRC and CAS led to significant prolongation in survival (*P* < 0.05) and reduction in residual fungal burden (*P* < 0.05) compared with CAS alone, and decrease in serum galactomannan levels (*P* < 0.05) compared with either agent alone. The survival in the combined therapy groups was significantly improved compared to VRC monotherapy for the strains of *A. flavus* and *A. niger* (*P* < 0.05), but no significant difference for the strains of *A. fumigatus* (*P* > 0.05).

**Conclusions:**

Combination of VRC and CAS was synergistic in IPA by *A. flavus* and *A. nige*r, but small efficacy benefits in IPA by *A. fumigatus*.

## Introduction

Invasive aspergillosis (IA) is an opportunistic infection caused by the fungi of the genus *Aspergillus*. Over 90 % of IA cases involve the lung, leading to invasive pulmonary aspergillosis (IPA) [1]. *Aspergillus fumigatus* is the most common species recovered from cases of IPA [2], followed by *Aspergillus flavus* and *Aspergillus niger*. Liking with *A. fumigatus*, IPA caused by *A. flavus* or *A. niger* is also associated with high mortality rates [3].

Voriconazole (VRC) is considered the primary therapy for IPA, based on the results of randomized clinical trials [4, 5], alternatives being liposomal amphotericin B (L-AMB), amphotericin B lipid complex (ABLC), caspofungin (CAS), micafungin, posaconazole, and itraconazole. Despite these treatment options, the outcomes of IPA remain poor, with mortality rates of 25–35 % 12 weeks after diagnosis [6]. Owing to the target of triazoles at cell membrane and echinocandins at cell wall [4], the combination therapy of azoles and echinocandins may result in synergistic interaction against *Aspergillus* spp, including a wider spectrum of efficacy, lowered toxicity, and prevented the emergence of resistance [7–9]. However, some studies showed that the combination of azole and echinocandin could not significantly improve the therapeutic outcome [10], even may be potentially antagonistic [11].

There are some reports of combination therapies of azole and echinocandin in the treatment for IA infected by *A. fumigatus* [7–14],more rarely by *A. flavus* [15] and *A. niger*. In this study, we established the model of IPA in transiently neutropenic rats infected by *A. fumigatus*, *A. flavus,* or *A. niger.* The dosage of VRC and CAS was adjusted according to the pharmacokinetic/pharmacodynamic (PK/PD) described in the previous studies [10, 12, 16–18]. The therapeutic efficacy and difference in both agents in every strain, administered alone or in combination, were examined.

## Materials and Methods

Three clinical isolates of *A. fumigatus,* *A. flavus,* and *A.* *niger* obtained from patients with proven IPA in Jinling hospital were used in this study. The isolates had been stored in 10 % glycerol broth at -80 °C. To prepare the inocula, the isolates were cultivated on Sabouraud dextrose agar (SDA) at 35 °C for 5 days and cultures were then suspended in sterile 0.1 % Triton 80 in phosphate-buffered saline (PBS) and filtered through sterile gauze to remove hyphae. The resulting suspensions were adjusted to the desired concentration of 1 × 10^9^ conidia/mL in sterile PBS by counting with a hemacytometer. The conidial suspension was used within 24 h and stored at 4 °C.

### In Vitro Studies

The in vitro antifungal susceptibility test of the three strains to VRC (Vfend, Pfizer Inc. Madrid, Spain) and CAS (Cancidas, Merck Sharp & Dohme Pty. Ltd. New South Wales, Australia) was performed in triplicate according to the Clinical and Laboratory Standards Institute (CLSI) standard M38-A2 microdilution methods [19]. For VRC,the minimal inhibitory concentration (MIC) was determined, and for CAS, the minimal effective concentration (MEC) was determined. The interactions of the combination of VRC and CAS were evaluated by determining the fractional concentration index (FICI) using a checkerboard method [20]. MIC end points were determined as MIC-0 (100 % of growth inhibition) of VRC alone and in combination with CAS. The FICI was defined as FICI = (Ac/Aa) + (Bc/Ba), where Ac and Bc are the MICs of VRC and CAS in combination, Aa is the MIC of VRC, and Ba is the MEC of CAS, as previously described [21–23]. Drug interactions were classified as synergistic (FICI ≤ 0.5), indifferent (FICI > 0.5 but ≤ 4), or antagonistic (FICI > 4) [24].

### Animal Model

A total of 180 male Sprague–Dawley rats, aged 6–8 weeks, weight 220–250 g, were used in these experiments. All the animals were housed under standard conditions and allowed ad libitum access to food and water. This study was conducted in conformity with institutional guidelines for the care and use of laboratory animals in Jinling Hospital, Nanjing, China, and performed according to the National Institutes of Health Guide for Care and Use of Laboratory Animals. Pulmonary aspergillosis was established as described elsewhere [25]. The animals were immunosuppressed with cyclophosphamide (75 mg/kg of body weight intraperitoneally twice) plus Methylprednisolone (13 mg/kg intramuscularly once) 2 days before infection. And the animals received Levofloxacin (Cravit; 10 mg/kg intravenous injection daily) from day 2 preinfection to day 10 after challenge to prevent bacterial infections. Rats were intratracheally inoculated with a single administration of 1 × 10^8^ conidia of *A. fumigatus*, *A. flavus,* or *A. niger* in 100 μl of sterile PBS [26].

### Antifungal Treatment

The efficacy of combination therapy using VRC and CAS was assessed in this study. For each strain and each treatment, 180 animals were randomized into groups of 15 rats. Ten rats were assigned randomly to the survival study and five rats for residual fungal burden and histopathological studies. Rats were grouped to receive VRC 10 mg/kg q12 h intravenously, CAS 1 mg/kg/day intravenously, a combination of VRC(10 mg/kg q12 h intravenously)and CAS (1 mg/kg/day intravenously), or no drug (untreated controls). All treatments began 1 day post-infection and continued for 10 days.

### Survival Study

Ten rats were assigned randomly to the survival study. The survival time after infection was recorded daily for 15 days.

### Galactomannan Assays

Blood from each rat was collected every other day to determine serum galactomannan levels. Serum galactomannan concentrations were performed by the Platelia Aspergillus EIA (Platelia Aspergillus; Sanofi Diagnostics, Marnes-La Coquette, France) according to the manufacturer’s instructions. Values of EIA were expressed as galactomannan index (GI) plotted over time.

### Residual Fungal Burden

The rats were killed on day 5 after challenge. Each right lung, weighed individually, placed (W:V = 1:1) in sterile 0.1 % Triton 80 in PBS, and homogenized in a tissue homogenizer. Serial tenfold dilutions of the homogenates were cultured for 48 h on SDA plates at 35 °C. Fungal burden was expressed as log 10 CFU/g of lung tissue.

### Histopathological Study

The left lungs of the killed rats were excised and fixed in 10 % buffered formalin. Paraffin-embedded tissue sections were then sectioned and stained with hematoxylin–eosin (HE).

### Statistical Analysis

Statistical evaluation of survival was done by the Kaplan–Meier analysis. Differences in rat survival rates were assessed by the log rank test. Galactomannan serum levels and fungal burden data were assessed by the Mann–Whitney U test. A *P* value of ≤ 0.05 was considered to be statistically significant.

## Results

### In Vitro Studies

The in vitro antifungal activity of VOR and CAS alone or in combination against the three strains of *Aspergillus* is shown in Table 1. The MIC for VOR was 0.5 μg/ml for the isolate of *A. fumigatus* and 0.25 μg/ml for both isolates of *A. flavus* and *A. niger*. The MEC for CAS was 0.06 μg/ml for both isolates of *A. fumigatus* and *A. flavus,* and 0.03 μg/ml for the isolate of *A. niger.* FICI indices ranged from 0.25 to 0.50, indicating the combination of VOR and CAS synergy for each strain.

**Table 1:** In vitro antifungal activity of voriconazole and caspofungin against three strains of *A. fumigatus, A. flavus,* and *A. niger*

Strain	MIC–VRC(μg/ml)	MEC–CAS(μg/ml)	FICI
*A. fumigatus*	0.5	0.06	0.50
*A. flavus*	0.25	0.06	0.38
*A. niger*	0.25	0.03	0.25

*VRC* voriconazole, *CAS* caspofungin, *MIC* minimal inhibitory concentration, *MEC* minimal effective concentration, *FICI* fractional inhibitory concentration index

### Survival

**Fig. 1:**
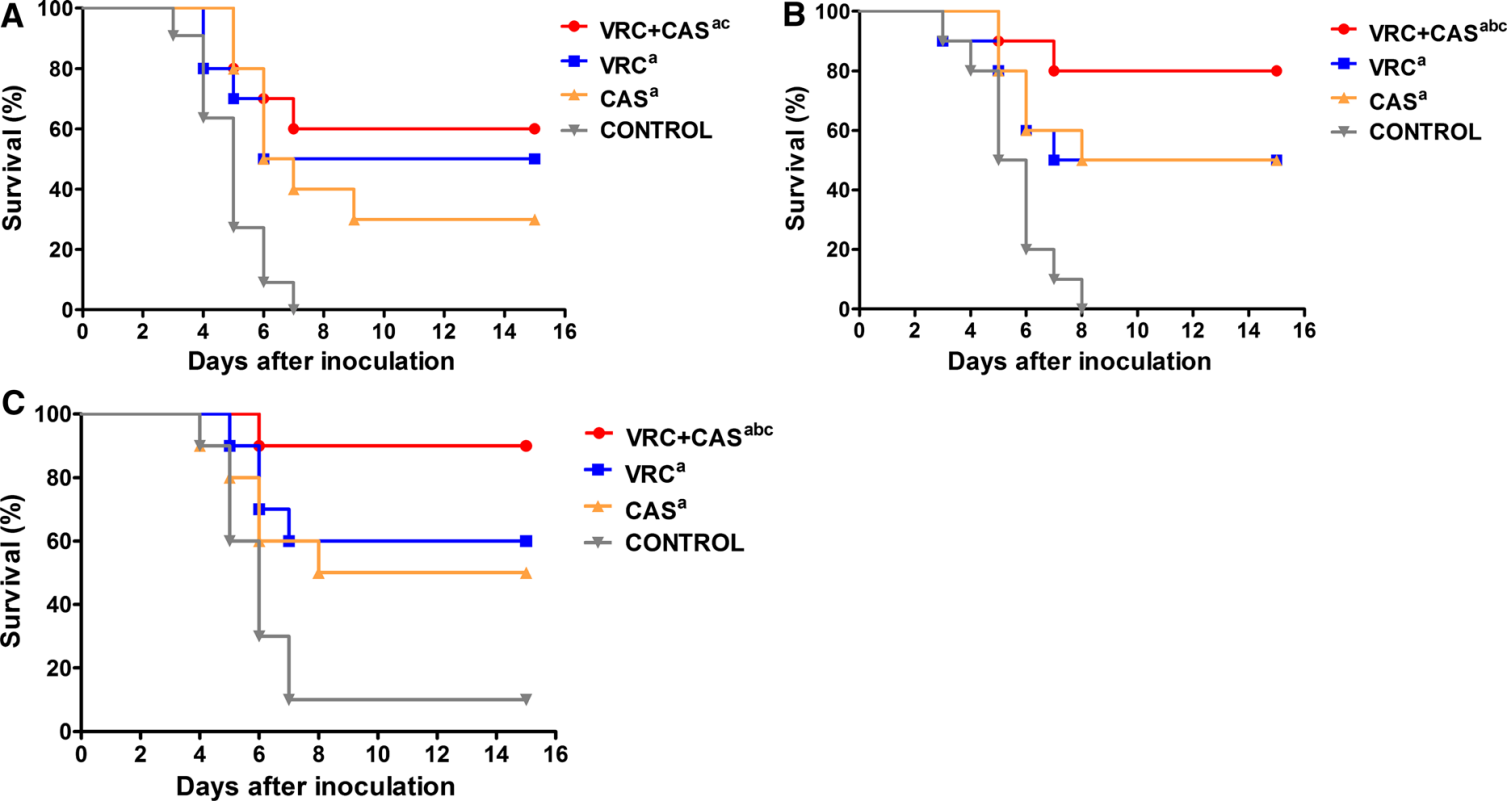
Survival of rats infected with 1 × 10^8^ conidia of *A.fumigatus* (**a**), *A. flavus* (**b**) and *A. niger* (**c**). VRC, voriconazole at 10 mg/kg q12-h intravenous; CAS, caspofungin at 1 mg/kg/day intravenous. All treatments began on day 1 post-infection and continued for 10 days. a *P*  < 0.05 versus CONTROL; b *P* < 0.05 versus VRC; c *P* < 0.05 versus CAS

Survival was prolonged among the rats treated with the combination of VRC and CAS for all the strains (Fig. 1.). The survival of rats in the combined therapy groups was significantly improved compared to the CAS monotherapy for the three strains tested (*P* < 0.05), and compared to VRC alone for the strains of *A. flavus* and *A. niger* (*P* < 0.05). Even though the survival rate of the combined therapy is higher than the VRC monotherapy for the strain of *A. fumigatus*, there were no significant differences (*P* > 0.05).

### Serum Galactomannan

**Fig. 2:**
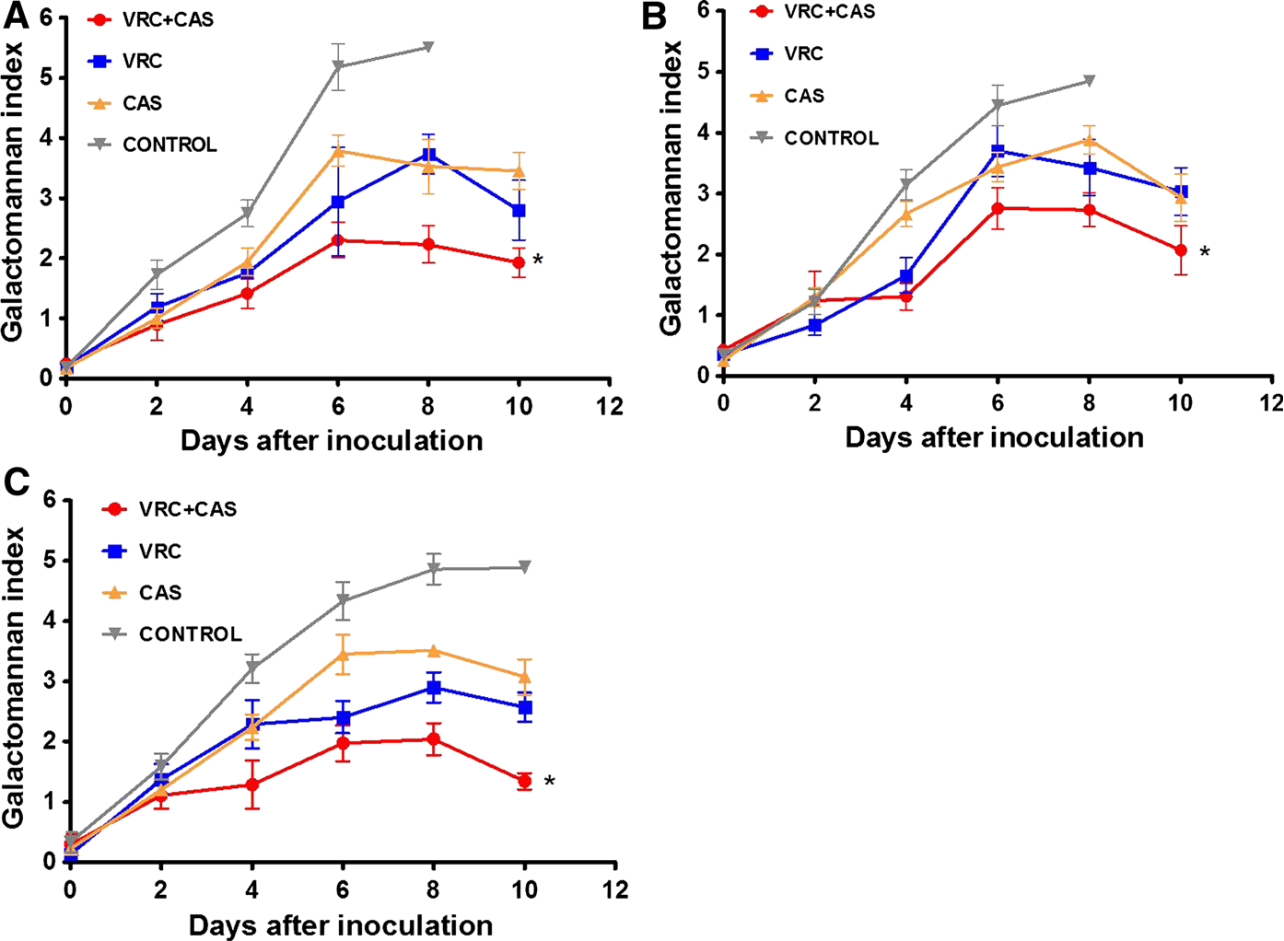
Expression of galactomannan in rats infected by *A. fumigatus* (**a**), *A. flavus* (**b**) and *A. niger* (**c**). Animals treated with the VRC and CAS combination had significantly lower levels of galactomannan in comparison with all other groups (*, *P* < 0.05). VRC, voriconazole at 10 mg/kg q12-h intravenous; CAS, caspofungin at 1 mg/kg/day intravenous

The serum galactomannan levels were significantly lower in the rats combined therapy of VRC and CAS in comparison with the other groups for all the strains (*P* < 0.05) (Fig. 2). There was no difference in VRC groups and CAS groups in comparison with the control groups (*P* > 0.05). However, the levels of galactomannan were significantly lower on day 6 and day 8 in VRC groups and CAS groups compared to the control groups (*P* < 0.05).

### Residual Fungal Burden

**Fig. 3:**
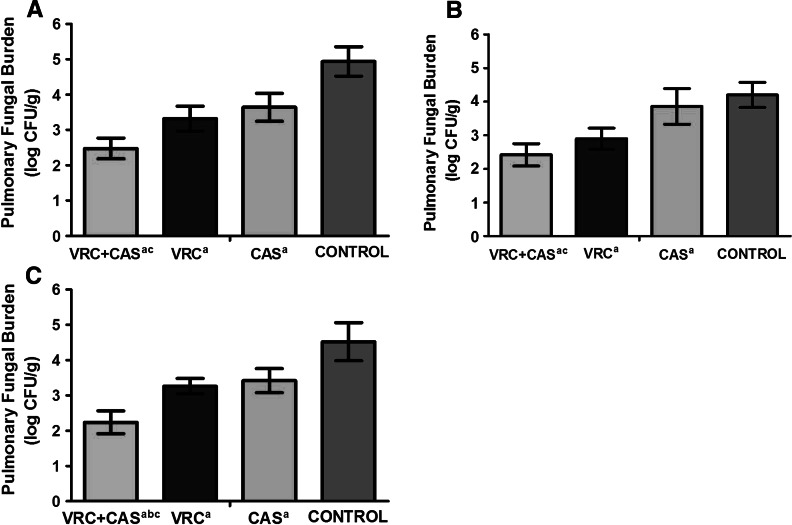
The mean pulmonary tissue residual fungal burden (log CFU/g) in rats infected with A. fumigatus (**a**), *A. flavus* (**b**) and *A. niger* (**c**) on day 5 after challenge. *VRC*, voriconazole at 10 mg/kg q12 h intravenous; *CAS*, caspofungin at 1 mg/kg/day intravenous. *a* *P* < 0.05 versus *CONTROL*; *b* *P* < 0.05 versus VRC; *c P* < 0.05 versus CAS

The results of the pulmonary tissue residual fungal burden are shown in Fig. 3. There was significant reduction of residual fungal burden (CFU/g) in the combination of VRC- and CAS-treated rats, compared with the effect of CAS alone, or no treatment for all the strains (*P* < 0.05). The combined therapy worked better than VRC in only the strain of *A. niger* (*P* < 0.05). There were no significant differences in the combination rats in comparison with that of VRC alone for the strain of *A. fumigatus* and *A. flavus* (*P* > 0.05).

### Histopathology

**Fig. 4:**
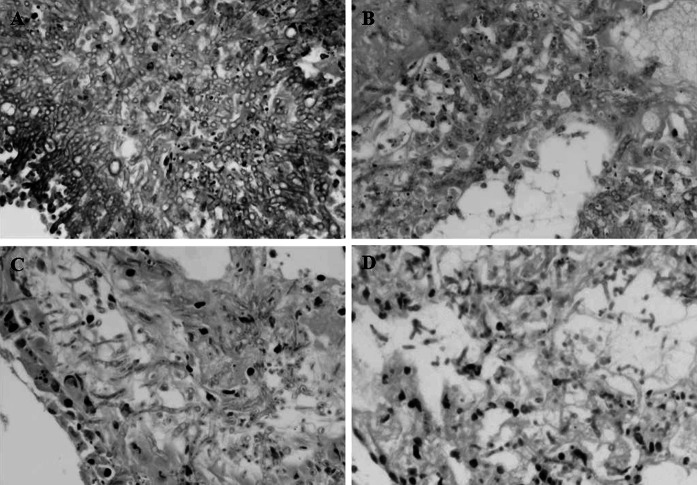
The histological studies in rats infected with A. fumigatus (**a**), *A. flavus* (**b**), and *A. niger* (**c**) on day 5 after challenge. **a** Lung sections of control rat infected with the *A. fumigatus*; HE × 200. **b** Lung section of a rat infected with the *A. fumigatus* treated with CAS; HE × 200. **c** Lung section of a rat infected with the *A.* *fumigatus* treated with VRC; HE × 200. **d** Lung section of a rat infected with the *A. fumigatus* treated with VRC and CAS; HE × 200

The histological features of aspergillosis were studied in the lungs of rats in all treatment groups (Fig. 4). Histological studies of untreated control rats showed dense clumps of hyphae and demonstrated a typical acute angle branching septate hyphae *A. fumigatus.* Rats treated with CAS showed alveolar collapse, inflammatory infiltration, and signs of necrosis, which were relatively mild in rats treat with VRC and the combination of VRC and CAS.

## Discussion

In this study, we investigated the efficacy of the combination of VRC and CAS compared to both monotherapies in vitro and in vivo using an experimental model of IPA infected by *A. fumigatus*, *A. flavus,* or *A. niger*. The combination of VRC and CAS demonstrated significantly enhanced efficacy for the strain of *A. flavus* and *A. niger*. We observed an synergistic interaction in vitro for each strain,a prolongation of survival,a significant reduction in serum galactomannan levels, and residual fungal burden (CFU/g) in combination of VRC and CAS compared with drug alone or no treatment.

To our knowledge, this is the first study to evaluate the therapy efficacy of the echinocandin–triazole combination in transiently neutropenic rats infected by *A. fumigatus*, *A. flavus,* or *A. niger* as measured by the prolongation of survival, reduction pulmonary tissue residual fungal burden, decrease in serum galactomannan levels, and histological studies. However, the dose of VRC or CAS used and the species of Aspergillus assayed in this study were single, which were possible limitation of the experimental design. For the VRC, metabolized faster in rodents than in humans, the area under the concentration–time curve (AUC)/MIC ratio is considered the pharmacokinetic/pharmacodynamic (PK/PD) index determining therapeutic efficacy [10, 18]. For the echinocandins, such as CAS,both the AUC/MIC and the *C*_max_/MIC are considered the PK/PD index predicting efficacy [10, 12]. Van de Sande et al. [10] elucidated the pharmacokinetics of VOR in rats model of IPA and found that the dosage of VRC at 10 mg/kg q12 h showed excellent efficacy, without toxic side effects in renal and hepatic functions. Meanwhile, several studies showed that the dose of CAS at 1 mg/kg/d has a better therapeutic effect in treatment IPA [12, 16, 17]. So, there was only a single dose of VRC or CAS used in this study. Though, as previously described [7, 10, 11, 13], we used one sensitive isolate of each of the species of *Aspergillus* assayed, this may be a limitation of the study.

There are contradictory results in the previous studies VRC combined echinocandin treatment for invasive aspergillosis (IA). Van de Sande et al. [10], in a neutropenic rat model of IPA by *A. fumigatus*, found combining of VRC and anidulafungin (AFG) does not significantly prolong the survival compared to the VOR monotherapy(*P* = 0.3290). Petraitis et al. [13] demonstrated that the survival of persistently neutropenic rabbits of IPA by *A. fumigatus* treatment with VRC (10 mg/kg every 8 h) combined with AFG at 5 mg/kg/day was 60 %, but 27 % for the VRC + AFG (10 mg/kg/day) group (*P* < 0.001). It suggested that AFG at a dosage of 5 mg/kg/day was synergistic but antagonistic at 10 mg/kg/day. In our study, combining both agents worked better than CAS alone in each strains and better than VRC in the strains of *A. flavus* and *A. niger*. Similarly, the therapy with VRC and CAS combined in guinea pig models of IA by *A. fumigatus* significantly improved survival and achieved significant reduction in residual fungal burden compared to respective monotherapies [16]. In other studies [15], the combined treatment of VRC and AFG in a murine IA model infected by *A. flavus* was found to significantly improve the survival, reduce the fungal burden and the galactomannan levels in comparison with AFG alone, but significantly improved only in a few cases compared to VRC alone. In the in vitro studies, our results showed each *Aspergillus* was susceptible for VRC and AFG and synergistic effect of VRC combined with AFG. Seyedmousavi et al. [23], in a non-neutropenic murine model of IA by VRC-susceptible or VRC-resistant *A. fumigatus*, demonstrated that the combination of VRC and AFG was synergistic in VRC-susceptible IA, but additive in VRC-resistant IA. Comparison with the previous studies, there are certain discrepancies. These might be due to the deviation of the type and number of animals, infection route and dose, therapeutic dosage, and so on.

Our results demonstrated VRC monotherapy is more therapeutically effective than the use of CAS in the rats of IPA by *A. fumigatus*. Combination of VRC and CAS does not significantly improve the survival in the treatment for experimental IPA by *A. fumigatus.* While in the rats of IPA by *A. flavus* or *A. niger,* our data confirm the therapy of VRC and CAS significantly improve the therapeutic outcome compared with drug alone. It suggests that the efficacy of the combination is different between *Aspergillus* species and the efficacy in *A. flavus* and *A. niger* is better than in *A. fumigatus*. However, there are some studies of combination therapy for IPA by *A. fumigatus* [7, 10, 12–14], more rarely by *A. flavus* [15, 27] and *A. niger* [27, 28]. The therapeutic outcome of the studies or cases of IPA by *A. flavus* or *A. niger* are generally successful.

In conclusion, there is a synergistic effect of the combination of VRC and CAS in the treatment for IPA by *A. flavus* and *A. niger*. Meanwhile, our data indicate that the echinocandin–triazole combination in comparison with single agents may confer a small efficacy benefits in the treatment for IPA by *A. fumigatus*. The efficacy of the echinocandin–triazole combination for IPA by *A. fumigatus, A. flavus,* and *A. niger* needs further clinical studies.
